# PadelTracker100: A dataset for intelligent player and ball tracking in padel sports

**DOI:** 10.1016/j.dib.2026.112546

**Published:** 2026-02-11

**Authors:** Roberto Bada-Nerin, Paula Rodríguez-González, Denis Kreibel, Víctor Teodoro, Oscar D. Pedrayes, Rubén Usamentiaga, Yago Fontenla-Seco, Pablo Ben-Leston, Sebastian Rodriguez-Trillo, Marcin Jędrzejowski, Nicolás Lozano-García, Franco Mosquera-Bonasorte

**Affiliations:** NTT Data, Spain

**Keywords:** Computer vision, Ball detection, Pose estimation, Shot event, Sport analytics, YOLO, ViTPose

## Abstract

Advancements in computer vision and deep learning have revolutionized sports video analysis, enabling automated and precise data labeling. However, these technologies rely heavily on high-quality annotated datasets, which are essential for training supervised learning models. This article introduces PadelTracker100, a large-scale fully annotated dataset for padel, a rapidly growing sport, captured in a professional setting. The dataset, derived from two matches of the 2022 World Padel Tour (WPT) Finals at 1920 × 1080@30, contains nearly 100,000 frames with a single standard camera angle to reduce occlusions and ensure clarity. Annotations include ball trajectory tracking, real-world player positions, player pose estimation and shot event recognition, categorized into six classes: backhand, forehand, smash, serve, dropshot, and other. Ball trajectory annotations were generated using a semi-automatic pipeline with iterative YOLO training. Pose estimation was carried out with ViTPose-L, selected after comparing various state-of-the-art models. Shot events were annotated for 40,135 frames. A thorough manual refinement process was applied, ensuring annotation quality trough all annotation types. The lack of pre-annotated datasets has significantly restricted large-scale match analysis and the development of automated techniques in padel, hindering the progress of AI-driven solutions tailored for the sport. By addressing this gap, this dataset serves as a comprehensive benchmark for padel, fostering advancements in various applications such as cross-sport analysis, injury prevention, tactical evaluation, ball trajectory modelling, and real-time video processing.

Specifications Table

**Table:** 

Subject	Sport Sciences
Specific subject area	*Briefly describe the specific subject area. Max 150 characters (without spaces).* Frame annotations of professional padel tennis videos for video analysis.
Type of data	Ball, pose estimation annotations, players coordinates on the court (.json), and shot event recognition annotations (.csv)
Data collection	The dataset was created using two publicly available videos from the 2022 World Padel Tour (WPT) Master Final, held in Barcelona, which capture both the men’s and women’s finals, each spanning approximately three hours. The videos were collected in MP4 format, with footage exclusively selected from the standard main view camera to minimize occlusions caused by panels and structural elements.
Data source location	Original video data was collected from YouTube videos, with the following data source: - World Padel Tour - Barcelona, Spain - Location: Palau Sant Jordi (41°21′48″N 2°09′09″E) The processed data is available in the Zenodo repository
Data accessibility	Repository name: PadelTracker100: A Dataset for Intelligent Player and Ball Tracking in Padel Sports License: Creative Commons Attribution 4.0 International (CC BY 4.0) Data identification number: 10.5281/zenodo.17020011 Direct URL to data: https://doi.org/10.5281/zenodo.17020011
Related research article	*None*

## 1 Value of the Data

• **Enhanced Understanding of Player Mechanics**: This dataset provides detailed annotations of player body poses and court position, offering a crucial resource for analyzing the biomechanics of padel tennis. It enables precise studies of movement patterns, posture, and technique, leading to definitive insights into injury prevention strategies and the development of targeted training methods that enhance athletic performance while minimizing injury risk.
• **Ball Trajectory Analysis**: With precise ball position data in each frame, the dataset supports comprehensive analysis of ball trajectories. This allows for an in-depth understanding of the physics behind various shots, the optimization of shot placement strategies, and the development of predictive models for ball movement, directly benefiting coaching and gameplay analytics.
• **Shot Classification and Strategy Development**: The dataset’s inclusion of shot types enables precise classification and analysis of different shot techniques in padel tennis. This information is essential for developing strategic models, refining player decision-making processes, and designing effective training regimens focused on specific shot techniques.
• **Machine Learning and Computer Vision Applications**: The annotated data serves as a robust foundation for researchers in machine learning and computer vision to develop algorithms for automatic recognition and analysis of sports movements. This facilitates advancements in real-time tracking systems, automated video analysis, and enhanced broadcasting features for sports events.
• **Cross-Sport Comparative Studies**: This dataset serves as a fundamental resource for conducting comparative studies between padel tennis and other racket sports, such as tennis or squash. It enables the identification of the unique characteristics of padel tennis, understanding its specific demands on athletes and applying successful techniques and training methods across different sports to improve overall athletic health and performance.
• **Educational and Training Tools Development**: The dataset can be utilized to create educational and training tools for coaches and players. By providing visual and data-driven feedback on player performance and shot execution, these tools can enhance learning experiences, facilitate skill development, and promote evidence-based coaching practices in padel tennis.

## 2 Background

In recent years, padel, a modern racquet sport played in doubles, has demonstrated remarkable global growth in popularity. This expansion has driven demand for advanced techniques to enhance player performance, optimize training strategies and mitigate biomechanical injuries.

Recent advancements in computer vision and machine learning, have created new possibilities for automating these techniques in various sports [1]. In racquet sports, these methods have been applied to tasks such as player position detection [1,2], ball tracking [3,4], shot recognition [5,6] and visual tactical analysis [7,8]. Despite advancements, video analysis for padel faces significant challenges due to the enclosed court and structural elements causing occlusions that complicate those tasks.

These state-of-the-art approaches rely on deep learning and convolutional neural networks, which necessitate accurately labelled datasets [9]. Although datasets for general sports are available [10], specific datasets for padel are scarce, with a notable exception being Dominguez et al [11] who used deep learning for shot classification with wristband data and Javadinha et al [10] who used motion captured data.

The absence of pre-annotated datasets significantly limits large-scale match analysis and the advancement of automated techniques in padel. This shortage of data hinders the advancement of AI-driven solutions specifically designed for the sport.

## 3 Data Description

### 3.1 Data format and file structure

The PadelTracker100 dataset [12] consists of annotations for ball position, player position, shot detection and recognition, and player pose estimation at the frame level extracted from two padel matches. The source video files for this dataset consists of two full-length clipped videos from the padel matches, the male and female finals of 2022 World Padel Tour in Barcelona, where the used frames for annotation can be extracted. All videos are recorded in 1920 × 1080 resolution at 30 fps, ensuring consistent quality across the dataset. The videos are not stored in the dataset.

**Fig. 1:**
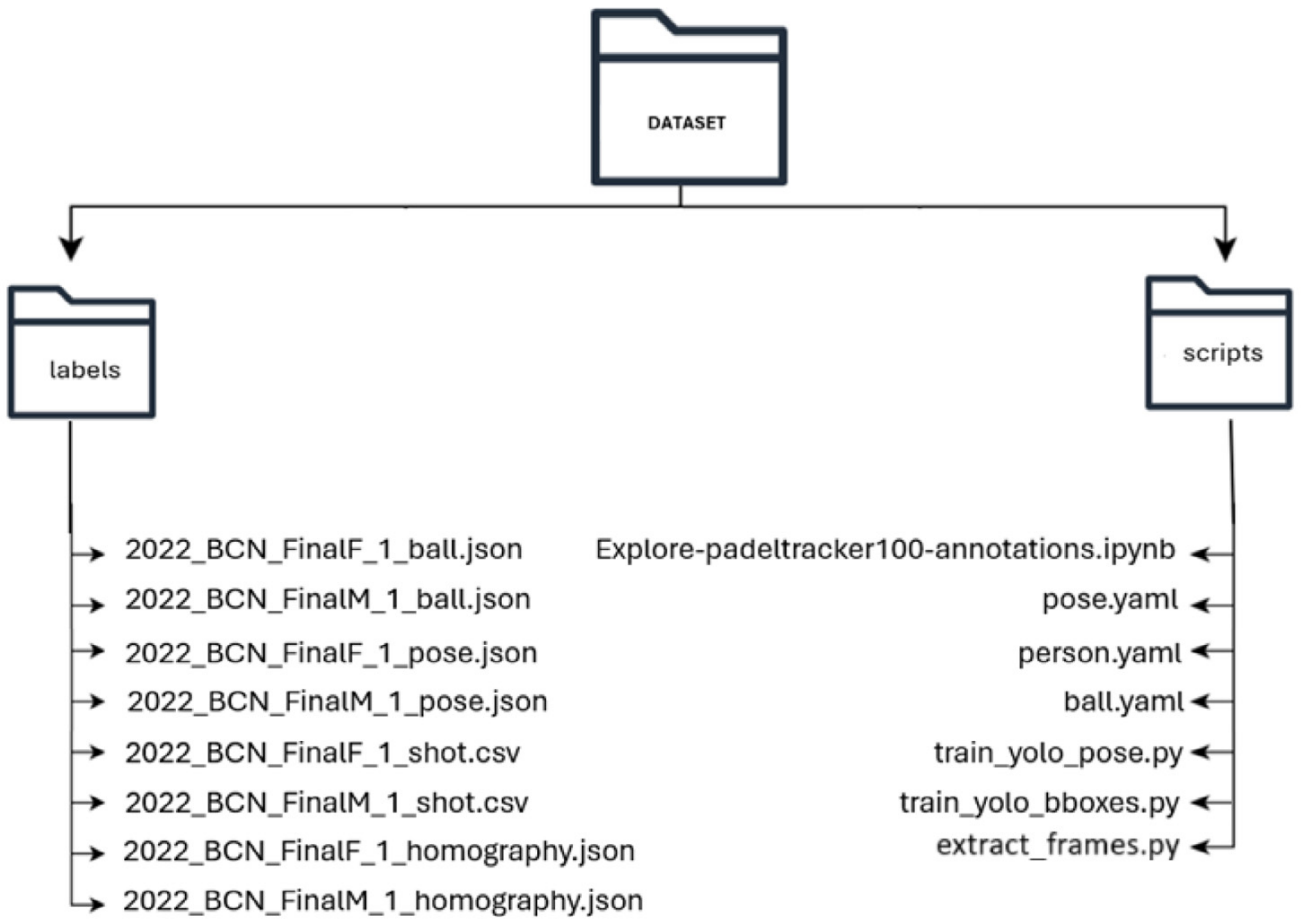
PadelTracker100 file structure.

The structure of this dataset is outlined in Fig. 1. All labelled data follows the Creative Commons Attribution 4.0 International (CC BY 4.0) license and can be found in the designated directory, where each file name corresponds to a specific video.

### 3.2 Project structure

**Scripts**: Includes utility scripts for extracting frames from the original videos, along with minimal training examples for YOLO models.

**Fig. 2:**
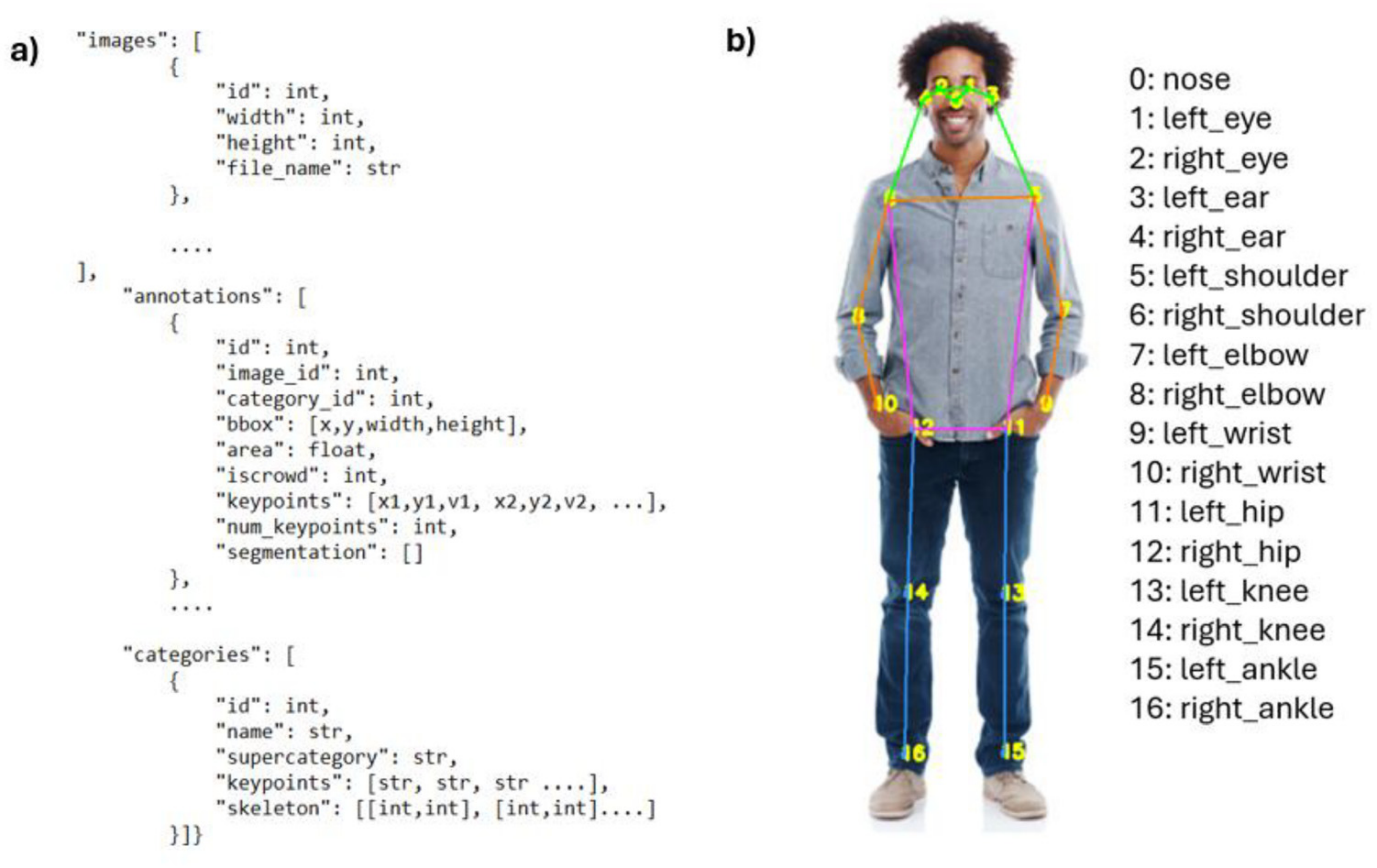
COCO Format. a) Structure of JSON annotation files for pose estimation. Ball annotations follow the same structure, excluding the keypoints, num_keypoints, and skeleton fields; b) Naming and location of keypoints,.

**Labels:** The annotations for ball detection, player position and pose estimation are stored in separate JSON files for each match, following the standardized COCO format for object and keypoint detection. This structure can be seen on Fig. 2**.** Shot events are stored as CSV files for each match. A data dictionary is available in APPENDIX: DATA DICTIONARY at the bottom of this document.

• **Ball Detection**: For ball detection, the bounding box around the ball in each frame is annotated. Each annotation includes the coordinates (x, y) and dimensions (width, height) of the bounding box.
• **Pose Estimation**: In pose estimation, the annotation process involves creating or representing skeletons based on the different keypoints detected for each person. The pose estimation annotations include the positions of the players on the court, represented by their bounding boxes (x, y, width, height). Additionally, each detected player has 17 keypoints annotated on their body, such as the nose, eyes, ears, shoulders, elbows, wrists, hips, knees, and ankles. These keypoints, which follow the COCO keypoint format and nomenclature, are represented by the coordinates (x1, y1) and visibility (v1), where ``v1'' indicates the visibility of each keypoint. The location and naming of the keypoints is also shown in Fig. 2.
• **Homography-projected court positions**: The real-world coordinates of players are stored in JSON files containing the homography transformation results of each frame (image_id) with all its players (id). Each player has its coordinates in meters (x, y) in a field called “location_m”.
• **Shot event annotations**: The shot event annotations are stored in CSV files, one for each match, each containing multiple columns. These files include the following information:
  ○ **Frame**: The name of the frame, formatted as frame_xxxxxx.PNG.
  ○ **Shot**: A binary flag indicating whether a shot occurs in the corresponding frame (1 for a shot, 0 for no shot). Frames were labeled as ``shot'' within a defined neighborhood around the actual shot, determined by the movement of the racquet of the player performing the shot.
  ○ **Type**: A string specifying the type of shot. If the ``has_shot'' flag is 0 (indicating no shot), the ``Type'' column will also be labelled as 0. In this case, six different shot types were considered, representing the most relevant shots in padel:
    ▪ **Backhand**: A shot where the player strikes the ball with the racket held behind their body, typically using both hands, but sometimes with one hand. It is a common shot for returning balls that come to the player's non-dominant side.
    ▪ **Forehand**: A shot where the player strikes the ball with the racket held in front of their body, typically with one hand. It is one of the most common especially when the ball is coming to the player's dominant side.
    ▪ **Smash**: An overhead shot, often executed when the ball is high in the air.
    ▪ **Serve**: The shot used to start a point, aiming to land it within the opponent's service box.
    ▪ **Dropshot**: A delicate shot executed with minimal power, intended to make the ball land just over the net.
    ▪ **Other**: A category for shots that do not fit into the above definitions or lack clear classification.

### 3.3 Dataset statistics

The clipped videos served as the source for extracting frames used in the analysis. The women's final video has a total duration of 25 min and 31.13 s, comprising 45,934 frames, while the men's final video lasts 29 min and 58.43 s, with 53,953 frames. Both videos were recorded at a consistent frame rate of 30 frames per second at 1920 × 1080. A summary of the video details is presented in Table 1.

**Table 1:** Clipped video details and statistics.

	2022_BCN_FinalF_1	2022_BCN_FinalM_1
*Video File Format*	.mp4	.mp4
*Frame Resolution*	1920 × 1080	1920 × 1080
*Frames Per Second (FPS)*	30	30
*Frame Count*	45,934	53,953
*Length (hh:mm:ss.ms)*	0:25:31.13	0:29:58.43

The distribution of annotations for shots, balls, and players reveals distinct differences between the female and male finals, reflecting variations in play style and frequency (Tables 2 and 3).

**Table 2:** Female Final (2022_BCN_FinalF_1) statistics.

	Total Frames Containing The Annotation	Percentage (%)	Mean Annotation Density
***Total Frames***	*45,934*	*-*	*-*
***Shot annotations***	***20,100***	***43.7584***	***1.0***
*No-Shot*	*12,598*	*27.4263*	
*Forehand*	*2336*	*5.0856*	
*Backhand*	*1730*	*3.7663*	
*Smash*	*2020*	*4.3976*	
*Serve*	*843*	*1.8352*	
*Other*	*548*	*1.193*	
*Dropshot*	*25*	*0.0544*	
***Ball annotations***	***45,934***	***100***	***1.0***
*Ball*	*37,904*	*82.5184*	
*No-Ball*	*8030*	*17.4816*	
***Player annotations***	***45,934***	***100***	***4.0***
*Person*	*45,934*	*100*

**Table 3:** Male final (2022_BCN_FinalM_1) statistics.

	Total Frames Containing The Annotation	Percentage (%)	Mean Annotation Density
Total Frames	53,953	-	-
Shot annotations	20,035	37.1342	1.0
No-Shot	14,287	26.4805	
Forehand	1528	2.8321	
Backhand	1649	3.0564	
Smash	1204	2.2316	
Serve	891	1.6514	
Other	410	0.7599	
Dropshot	66	0.1223	
Ball annotations	53,953	100	1.0034
Ball	19,320	35.8089	
No-Ball	34,633	64.1911	
Player annotations	53,953	100	3.993
Player	53,888	99.8795	
No-Player	65	0.1205	

A comparative analysis of shot type distributions between the women’s and men’s finals highlights distinct gameplay patterns. “No-Shot” frames dominated both matches (27.4 % in the women’s final vs. 26.5 % in the men’s), but women displayed a greater reliance on offensive strokes, particularly forehands (5.1 % vs. 2.8 %) and smashes (4.4 % vs. 2.2 %). Backhand usage was also slightly higher in the women’s match (3.8 % vs. 3.1 %), while serves were nearly identical across genders (1.8 % vs. 1.7 %). Men, in turn, showed modestly higher use of “Other” and “Dropshot” categories (0.76 % and 0.12 %, respectively) compared to women (1.19 % and 0.05 %).

A chi-square test across all seven shot categories confirmed that these differences were highly significant (χ²(6) = 523.12, *p* < 0.001), with a Cramér’s V of 0.11 indicating a small but meaningful effect size. These results suggest that female players in this final favored a more offensive style centered on forehands and smashes, while male players adopted a more balanced distribution with slightly greater use of tactical variations.

The distribution of ball annotations also shows variation. In the female final, 37,904 frames (82.52 %) contain a ball, while 8030 frames (17.48 %) do not. In the male final, 19,320 frames (35.81 %) feature the ball, with 34,633 frames (64.91 %) lacking the ball. In terms of player annotations, all 45,934 frames in the female final include player annotations. Similarly, in the male final, 53,888 frames (99.99 %) have player annotations, with only 65 frames (0.12 %) lacking them. All this statistic information can be found on Table 2 and Table 3.

**Fig. 3:**
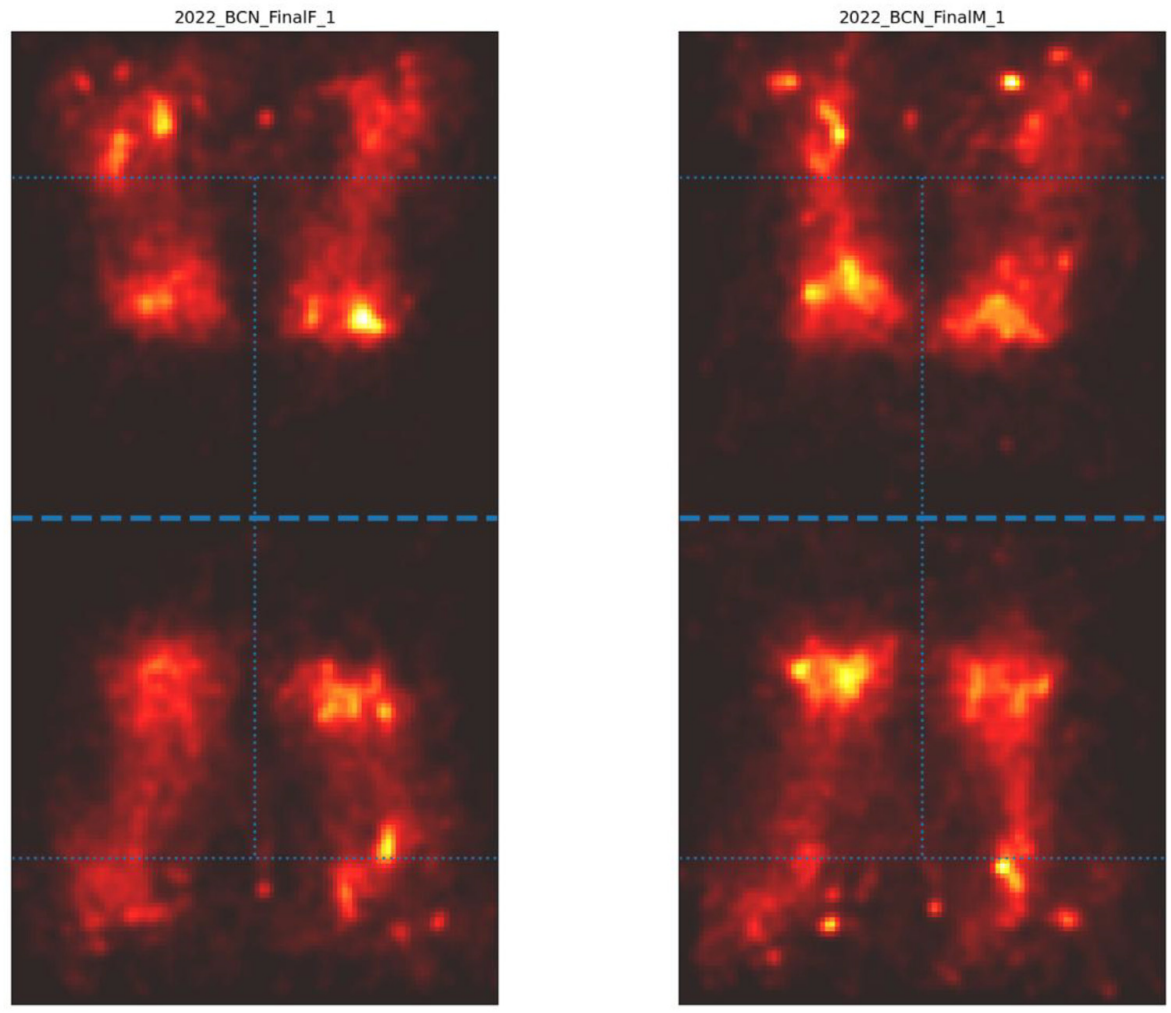
Homography position heatmaps of players in the female and male finals.

Spatial heatmaps of player positioning reinforce this interpretation (Fig. 3). In the women’s final, activity was concentrated around the service line and mid-court, consistent with frequent advances to the net and an aggressive tactical approach. In contrast, the men’s final showed broader intensity along the baseline and deep half-court, with weaker concentrations at the net, reflecting longer rallies and baseline-dominant exchanges. Together, the statistical and spatial analyses indicate that female players employed a net-oriented, pressure-driven style, whereas male players relied more on baseline control and extended rally construction.

## 4 Experimental Design, Materials and Methods

A step-by-step procedure has been followed to compile the dataset. Each of these steps is discussed in detail in this section.

### 4.1 Data acquisition

The raw videos collected from YouTube capture both the men’s and women’s finals, each spanning approximately three hours, totalling 6.1 GB in MP4 format. These matches were filmed from various camera angles, including views of the court, close-ups of players, and shots of the audience.

### 4.2 Data preprocessing

This diversity in camera angles introduces the need to preprocess the videos to extract only the relevant frames that align with the objectives of the dataset. In this dataset creation, only the main view camera—considered the de facto standard for padel matches—was deemed optimal for the task. This non-zenithal view minimizes the impact of occlusions caused by the mesh panel and structural elements. In this configuration, the mesh panel spans the region from the bottom of the net to near the opposite service line. This camera, positioned 7.6 m above the floor and 15.5 m behind the glass panels, captured video at a resolution of 1920 × 1080 and 30 frames per second.

As part of the pre-processing pipeline, the videos were filtered to retain only frames recorded from the standard main camera. For this task, Clipchamp [14], a video editing software, was used to extract the appropriate footage. This tool enabled precise trimming and preparation of the video, ensuring that only frames captured from the optimal camera angle were kept, while non-relevant segments, such as audience shots or alternate angles, were removed. For frame-level analysis, both clipped videos were uploaded to CVAT (Computer Vision Annotation Tool) [15], which automatically splits them into individual frames, making them ready for annotation. This step ensured that each frame could be accurately labelled and analysed for further processing.

### 4.3 Annotation methodology

#### 4.3.1 Ball annotation

**Fig. 4:**
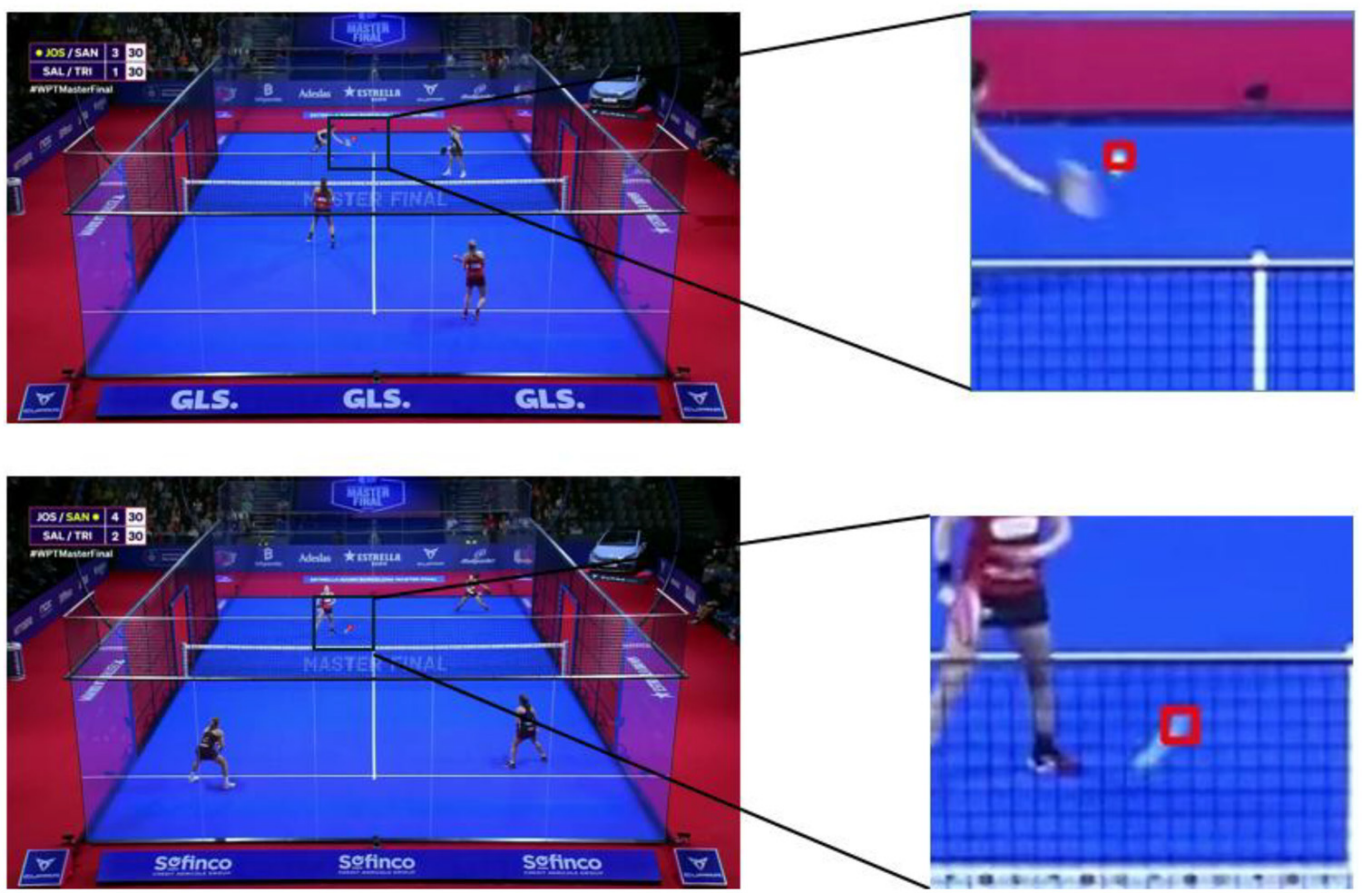
Ball annotations. Frames 16,223 and 23,627 of the woman’s finals.

In the case of the ball annotations, a semi-automatic annotation strategy was employed. Initially, 3000 frames were manually annotated and used as a training set for a YOLOv8 model [16]. It is important to note that the motion blur of the ball, caused by its high velocity and the low frame rate, often created the appearance of a continuous line in some frames. In such cases, the last visible point of the ball was recorded as its location (Fig. 4).

This manual annotation was followed by a three-step iterative approach in which successive YOLOv8 models were trained using annotations generated in the previous step. Training sets consisting of 3000, 12,000, and 40,000 frames were used for the first, second, and third iterations, respectively. The final model from this process was subsequently applied to annotate the remaining frames in the dataset. All three models were tested on a separate dataset of 379 frames that were not used in training or validation.

#### 4.3.2 Shot annotation

The shot annotation strategy consisted of manually labelling all the frames in a neighborhood around the shot. This neighborhood is defined by the movement of the racquet of the player that performed the shot. A total of 40,135 frames were annotated using this approach.

#### 4.3.3 Keypoint annotation

For the pose estimation process, a ViTPose-L model [17] was used. Again, a semi-automatic annotation process was carried out using this model, ensuring accurate tracking of keypoints across the dataset.

**Fig. 5:**
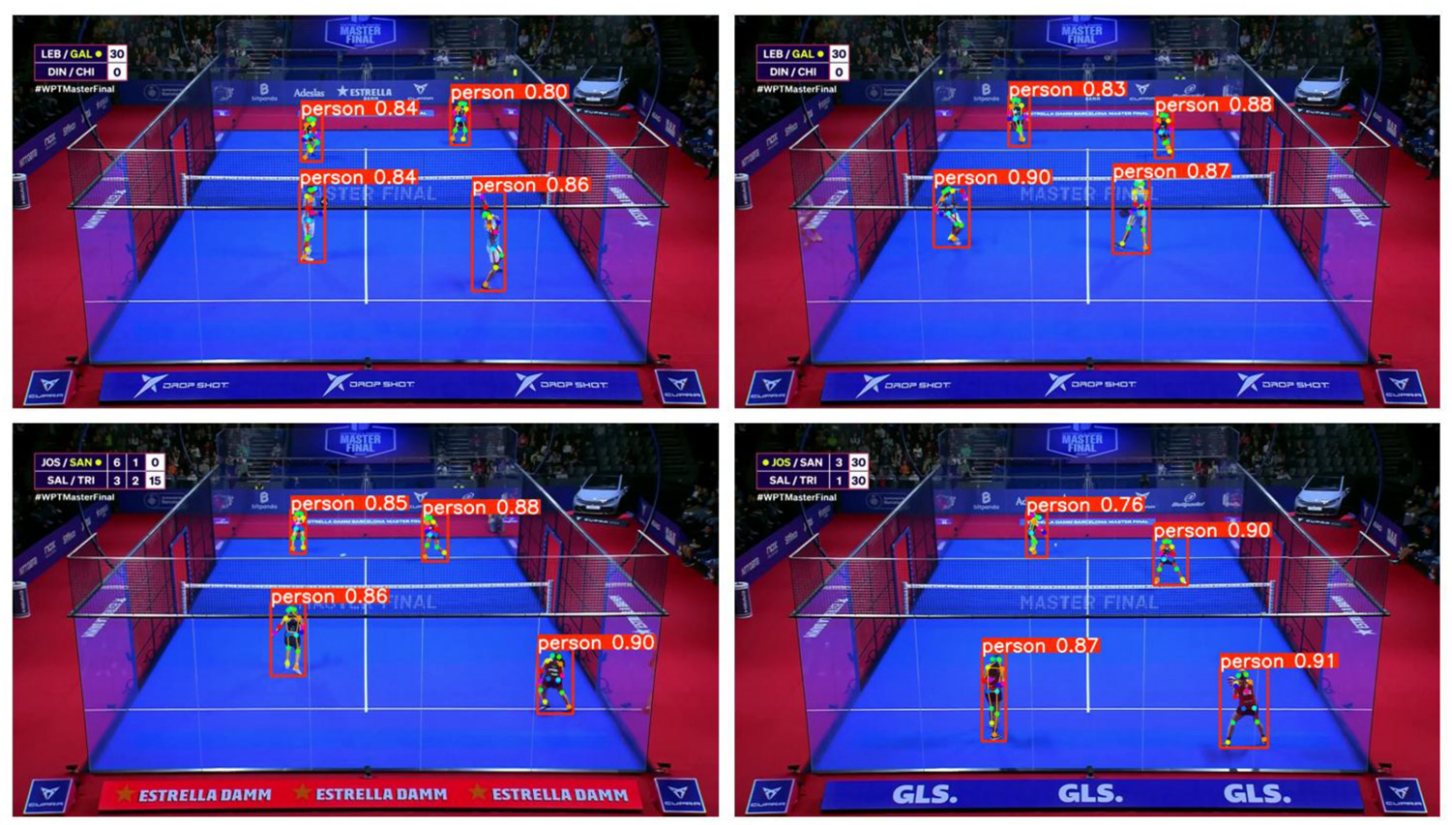
Pose estimation annotations from ViTPose-L model. Frames 001,178 and 001,008 of the man’s finals (top) and frames 37,861 and 15,370 of the woman’s finals (bottom).

This annotation process followed a multi-step approach. Initially, player detection was performed using a YOLOv8x model, which identified individual players in each frame. Only players detected with a confidence score greater than 0.7 were retained to reduce false positives, such as non-player entities like the audience or camera personnel. After detection, the ViTPose model was applied to estimate the 17 keypoints for each detected player. The resulting data was compiled into a JSON file containing predictions for both the bounding boxes and keypoints for each player (Fig. 5).

### 4.4 Refining the dataset

Once ball and keypoint predictions were generated by the models, the dataset underwent a manual review to ensure robustness and accuracy. Each annotator was assigned a portion of the frames and inspected them frame by frame, correcting errors where necessary. After this stage, all annotators collectively rewatched the full videos with the labels overlaid, providing a final quality check to guarantee annotation consistency and reliability.

In the final iteration, the players’ annotations achieved an inter-annotator agreement IoU of 0.826, while ball tracking reached 0.677. This discrepancy is primarily due to the blurriness of the ball caused by motion. To reduce this uncertainty, it was decided that the ball should consistently be labeled at the final position within the blur.

#### 4.4.1 Ball annotation refining

To ensure precise ball annotations, all predicted frames were meticulously reviewed, and any detected false positives were removed. The only exceptions to this were instances where the ball exited the camera's field of view, in which case the missing values were left unfilled.

#### 4.4.2 Keypoint annotation refining

To refine the pose estimation predictions, a thorough and time-intensive manual review process, led by experts, was implemented. After all frames from both videos were automatically annotated, a two-step process was conducted to ensure high-quality annotations.

The first step involved verifying the presence of all four players on the court in each frame, ensuring no players were missed and that no detections were mistakenly attributed to the audience or staff. The second step focused on the precision of keypoint locations for each detected player. This step required manually reviewing each keypoint and adjusting its position if necessary to align accurately with the player’s joint.

However, head-related keypoints (nose, eyes, and ears) were excluded from this refining task. These keypoints were deemed less relevant because they are often not visible in most frames—given that at least two players are typically facing away from the camera—and because they are less critical for future applications.

The outcome of this process was a thoroughly reviewed and fully annotated dataset comprising nearly 100,000 frames, each containing precise spatial positions of the players and accurate keypoint locations. These annotations were stored in the COCO format, ensuring consistency and compatibility with the dataset’s established standards.

#### 4.4.3 Homography transformation

To transform image coordinates into real-world court positions, a homography matrix was computed. This calibration process relied on manually selecting reference points corresponding to standardized features of the padel court, such as line intersections and corners. By mapping these points from image space to their real-world coordinates, a perspective transformation was established, enabling the accurate projection of pixels onto a two-dimensional representation of the court.

For player mapping, the average position of both ankle keypoints was used to approximate the player’s center of mass, providing a stable and consistent reference point across frames. This ensured that player positions were precisely aligned with the homography transformation and minimized noise from variations in individual keypoint detections.

**Fig. 6:**
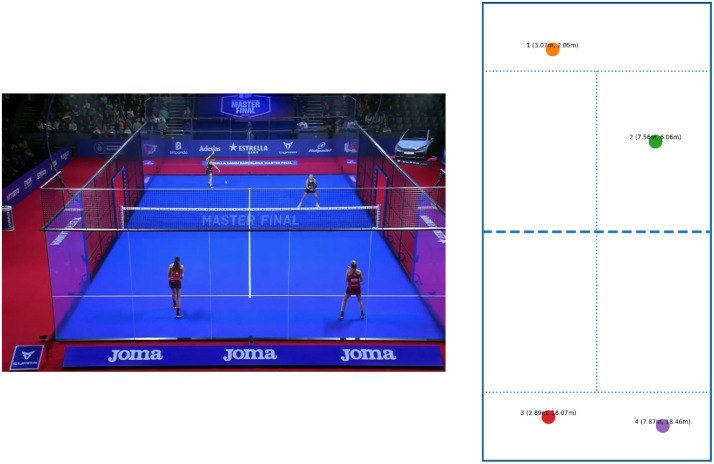
Homography transformation example.

Fig. 6 illustrates this procedure. The left panel shows the broadcast match footage, while the right panel depicts the homography calibration, where selected reference points have been assigned real-world measurements. Once applied, the homography allowed every pixel location in the annotated frames to be converted into metric distances, enabling quantitative analyses of player positioning, movement patterns, and ball trajectories within the actual dimensions of the padel court. This transformation ensures that spatial data extracted from video footage is both interpretable and comparable across matches, thereby enhancing the dataset’s utility for performance and tactical research.

## Limitations

The dataset has certain limitations that may affect its subsequent use and should be considered in future applications. Firstly, as the videos were collected from professional padel tournaments, they only involve professional players. This could create a generalization issue when applied to amateur matches, as the movements, tactics, and poses of amateur players often differ from those of professionals.

Moreover, the reliance on a single camera angle throughout the video introduces the risk of occluded poses or ball positions. This limitation could be addressed by incorporating additional camera angles in future datasets, providing a more complete and accurate representation of the players’ poses and ball trajectories. Other potential limitations include the lack of variation in playing conditions (e.g., lighting, court type), which could affect applications in real-world, uncontrolled environments. Incorporating a wider range of conditions, player types, and game scenarios would further enhance the generalizability of the dataset. Furthermore, the shot types in the dataset are imbalanced, as depicted in Table 2 and Table 3.

## Ethics Statement

The dataset is derived from publicly available videos on YouTube, specifically from the official World Padel Tour account. The data collection process adhered to YouTube's Terms of Service.

The original data belongs to the World Padel Tour [13], as they posted the videos on YouTube. The use of these video frames for research purposes complies with copyright laws. Since the dataset includes professional athletes performing in public sports events, anonymization of individuals is not necessary. The URLs to the original videos are provided, ensuring transparency and reproducibility.

The data collection process complied with YouTube’s scraping policies, as it was collected using automated tools that adhere to the platform's guidelines. The data collected for this study was obtained through an automated process using the YouTube API and Python web automation frameworks. No human subjects were involved in the data collection process beyond those appearing in publicly available videos. As the data is publicly available and features professional athletes, informed consent was not required for this study.

No conflict of interest exists in this submission. The authors declare that the work described in this paper is original and not under consideration for publication elsewhere, in whole or in part. Its publication is approved by all the authors listed.

## CRediT authorship contribution statement

**Roberto Bada-Nerin:** Conceptualization, Methodology, Software, Data curation, Writing – original draft. **Paula Rodríguez-González:** Methodology, Software, Data curation, Writing – original draft, Writing – review & editing. **Denis Kreibel:** Conceptualization, Methodology, Supervision, Project administration, Writing – review & editing. **Víctor Teodoro:** Conceptualization, Software, Supervision. **Oscar D. Pedrayes:** Conceptualization, Supervision, Project administration. **Rubén Usamentiaga:** Writing – review & editing, Resources. **Yago Fontenla-Seco:** Supervision, Writing – review & editing. **Pablo Ben-Leston:** Data curation. **Sebastian Rodriguez-Trillo:** Data curation. **Marcin Jędrzejowski:** Data curation. **Nicolás Lozano-García:** Data curation, Software. **Franco Mosquera-Bonasorte:** Writing – original draft.

## Figures and Tables

**Table:** 

Field Name	Data Type	Units	Description
id	Integer	N/A	A unique identifier for each frame/image
width	Integer	Pixels	The width of the frame, consistently 1920.
height	Integer	Pixels	The height of the frame, consistently 1080
file_name	String	N/A	The filename of the frame as “frame_XXXXXX.PNG”

**Table:** 

Field Name	Data Type	Units	Description
id	Integer	N/A	A unique identifier for the annotation instance
image_id	Integer	N/A	Corresponds to the id in the images object, linking the annotation to a specific frame.
category_id	Integer	N/A	Corresponds to the id in the categories object, identifying the object class (e.g., player or ball).
bbox	List [Float]	Pixels	The bounding box of the object, formatted as [x, y, width, height], where (x, y) is the top-left corner.
area	Float	Pixel^2^	The area of the bounding box (width * height)
iscrowd	Integer	N/A	A flag for crowd annotations; typically 0 in this dataset.
keypoints	List [Integer]	Pixels	**(Pose only)** A list of 51 values representing 17 keypoints [x1, y1, v1, x2, y2, v2, …]. Visibility v is 0 (not labeled), 1 (labeled but occluded), or 2 (labeled and visible). Commonly 2 in this dataset.
num_keypoints	Integer	N/A	**(Pose only)** The count of keypoints with visibility *v* > 0.
segmentation	List	N/A	[] in this dataset.
attributes	Dict	N/A	Details if the annotation is occluded and if the frame is a keyframe: {"occluded": false, ``keyframe'': false}

**Table:** 

Field Name	Data Type	Units	Description
id	Integer	N/A	A unique identifier for the category (e.g., 1).
name	String	Pixels	The name of the category (e.g., 'person', 'ball').
supercategory	String	Pixels	The parent category (e.g., 'person')
keypoints	List [String]	N/A	**(Pose only)** A list of the 17 keypoint names in order (e.g., 'nose', 'left_eye', etc.).
skeleton	List [List]	N/A	**(Pose only)** Defines the connections between keypoints to form a human skeleton.

**Table:** 

Field Name	Data Type	Units	Description
image_id	Integer	Pixels	Unique identifier of the source frame/image from which the homography was computed.
location_m	List [Id, List[Float]]	Meters	Mapping of players to their transformed positions in real-world court coordinates {id: [x, y] in meters)}.
location_px_raw	List [Id, List[Float]]	Pixels	Original raw pixel coordinates {id: [x, y]} of the players in the input image.
H	List [Float]	N/A	3 × 3 projective homography matrix (flattened) used to transform points from image pixel space to real-world meter space.
extent	List[Float]	N/A	Boundaries of the warped image in real-world coordinates, usually [xmin, xmax, ymin, ymax].

## Data Availability

ZenodoPadelTracker100: A Dataset for Intelligent Player and Ball Tracking in Padel Sports (Original data).

## Appendix: Contact

For any communication regarding this dataset, we provide an open contact email where users can send questions, feedback, or suggestions for improvement: PadelTracker100Dataset@gmail.com.

## Appendix: Data Dictionary

This appendix provides a detailed data dictionary of the primary objects contained in the dataset’s JSON files to facilitate adoption. The structure largely follows the COCO format (see references: https://cocodataset.org/#format-data and https://docs.aws.amazon.com/rekognition/latest/customlabels-dg/md-coco-overview.html), with the exception of the homography object. For clarity, the most relevant elements are explained below to support ease of use.
